# Perinatal maternal mental health and offspring internalizing and externalizing difficulties from early childhood through adolescence: Rhea mother—child cohort in Crete, Greece

**DOI:** 10.1007/s00787-025-02746-1

**Published:** 2025-05-21

**Authors:** Katerina Koutra, Chrysi Mouatsou, Katerina Margetaki, Theano Roumeliotaki, Georgios Mavroeides, Sofia Psoma, Mariza Kampouri, Marianna Karachaliou, Manolis Kogevinas, Lida Chatzi

**Affiliations:** 1 Department of Psychology, School of Social Sciences, University of Crete, Gallos Campus, 74100 Rethymno, Crete Greece; 2 Clinic of Ρreventive and Social Medicine, Department of Social Medicine, Faculty of Medicine, University of Crete, Heraklion, Crete Greece; 3 Institute of Environmental Medicine, Karolinska Institutet, Stockholm, Sweden; 4 Barcelona Institute for Global Health, Barcelona, Spain; 5 Hospital del Mar Medical Research Institute, Barcelona, Spain; 6 Department of Preventive Medicine, Keck School of Medicine, University of Southern California, Los Angeles, USA

**Keywords:** Maternal mental health, Internalizing symptoms, Externalizing symptoms, Attention deficit hyperactivity disorder, Trajectories

## Abstract

Longitudinal research assessing childhood mental health is limited, particularly in terms of tracking how early-life factors influence the development of emotional and behavioral disorders over time. The present study aimed to examine the influence of both antenatal and postnatal maternal mental health on children's emotional and behavioral development, with a focus on the trajectory of internalizing, externalizing and attention deficit hyperactivity disorder (ADHD) symptoms from early childhood through adolescence. The present study included 434 mother–child pairs of the Rhea mother–child cohort in Crete, Greece. Maternal depressive symptoms (EPDS), trait anxiety (STAI-Trait), and personality traits (EPQ-R) were assessed during the third trimester of pregnancy, while postnatal depressive symptoms (EPDS) were evaluated 8–10 weeks after childbirth. Children’s internalizing, externalizing and ADHD-related symptoms were assessed at ages 4 (using the SDQ and ADHDT, respectively), and at ages 6, 11, and 15 (using the CBCL and CPRS-R:S, respectively). Multivariate mixed regression models, incorporating a random intercept for each child and a random slope for age at follow-up, were used to analyze the trajectories of symptoms from ages 4 to 15. Significant long-term associations were found between maternal mental health and child outcomes between the ages of 4 and 15. Specifically, higher levels of maternal trait anxiety and neuroticism were associated with an increase in children's internalizing, externalizing, and ADHD symptoms. Both antenatal and postnatal maternal depressive symptoms were linked to a greater likelihood of internalizing and externalizing symptoms over time, but only postnatal depression was related to a rise in ADHD symptoms. The findings emphasize the importance of perinatal maternal mental health in children's emotional and behavioral development and suggest the need for prevention and early intervention programs to support the mental health of both mothers and children.

**Supplementary Information:**

The online version contains supplementary material available at 10.1007/s00787-025-02746-1.

## Introduction

Internalizing disorders, including anxiety and depression, and externalizing disorders, like conduct disorder and attention deficit hyperactivity disorder (ADHD) are among the most common mental health issues in children and adolescents, with prevalence rates around 10% and 20%, respectively [1, 2]. These disorders frequently co-occur leading to more severe and long-lasting symptoms [3]. Among externalizing problems, ADHD is the most prevalent and frequently diagnosed condition marked by enduring patterns of inattention, hyperactivity, and impulsivity [4].

The Developmental Origins of Health and Disease (DOHaD) framework emphasize the importance of antenatal and early life environments in influencing long-term health outcomes and disease risk [5], including mental health [6]. Evidence indicates that maternal distress during critical periods of brain development can lead to lasting changes in neurobiological processes and brain structure, increasing vulnerability to mental health disorders [7].

Maternal depressive symptoms during the perinatal period are common with antenatal and postpartum depression prevalence rates estimated at 28.5% and 27.6%, respectively [8]. Both antenatal and postnatal depressive symptoms have been linked to offspring internalizing and externalizing symptoms across all developmental stages. Specifically, studies have consistently associated antenatal depression with emotional and behavioral difficulties in toddlerhood [9], early childhood [10, 11], middle childhood and adolescence [12, 13]. Specifically for ADHD-related symptoms, antenatal depression has been associated with greater difficulties in childhood [14–16]. Furthermore, postnatal depression has been linked to internalizing and/or externalizing symptoms in toddlerhood [9, 17], early childhood [18], middle childhood [10, 19, 20], adolescence and adulthood [21]. Additionally, postnatal depression is strongly linked to increased ADHD symptoms across childhood and adolescence [22]. Limited evidence links postpartum depression to the developmental pathways of internalizing and externalizing symptoms from toddlerhood through childhood [23, 24], extending into adolescence [25] and continuing into adulthood [21].

Furthermore, studies have consistently shown that maternal anxiety during pregnancy is linked to increased internalizing and externalizing symptoms in offspring during toddlerhood [26, 27], early childhood [28], middle childhood [12, 29], and adolescence [30, 31]. Antenatal anxiety has also been linked to increased ADHD symptoms in children [15, 32, 33]. Longitudinal research shows that while maternal antenatal anxiety often co-occurs with depression and both are linked to negative developmental outcomes [34], antenatal anxiety can independently predict worse emotional and behavioral problems in offspring, even when accounting for depression [35].

Beyond maternal depression and anxiety, maternal personality may significantly influence the emotional and behavioral development of offspring, with Eysenck’s Giant Three personality model—comprising neuroticism, extraversion, and psychoticism—serving as a key framework in understanding these effects [36, 37]. Research indicates that higher maternal extraversion is associated with enhanced social-emotional development in toddlers, while higher neuroticism is linked to increased behavioral difficulties, including ADHD symptoms, in young children [15, 38]. Research has also linked maternal neuroticism to internalizing and externalizing symptoms in middle childhood and adolescence [39], but further investigation is needed to fully understand the role of maternal personality in children's outcomes.

Longitudinal research in this field is limited, with most studies focusing on short-term outcomes and lacking comprehensive follow-up across key developmental stages, creating a gap in understanding the extended influence of early-life experiences and maternal mental health on developmental trajectories. A more robust and comprehensive approach, with particular emphasis on the antenatal and postnatal periods, is crucial for elucidating the enduring impact of these early influences on childhood and adolescent mental health outcomes. Furthermore, this study is particularly important in Greece due to the country's unique socio-economic context, which has been marked by a series of significant crises. The 2008 financial crisis triggered widespread economic hardship, increasing poverty, social exclusion, and mental health challenges, which placed significant strain on both families and healthcare systems [40]. Research from this period indicated that around 10% of adolescents developed mental health problems during the recession, with food insecurity increasing their vulnerability, highlighting the significant negative impact of economic hardship on youth mental health [41]. The COVID-19 pandemic further exacerbated these issues, with widespread social isolation, disruptions to daily life, and increased parental stress contributing to higher psychological distress in children [42]. These events have had profound effects on the mental health and developmental trajectories of children and adolescents, making it crucial to examine how perinatal maternal mental health influences long-term child development in this context.

Within the context of a population-based mother–child cohort study in Crete, Greece (Rhea Study), we aimed to address whether perinatal maternal mental health associates with offspring’s developmental trajectories of internalizing and externalizing symptoms from early childhood through adolescence. While previous research in this population has linked poor maternal mental health to higher risk for postpartum depression [43], adverse birth outcomes [44] and delayed neurodevelopment [15, 38], the present study aims to provide a comprehensive, long-term understanding of developmental trajectories and how early maternal distress may impact children's mental health across key stages of development. Our specific objectives are to examine a) whether the relationship between perinatal maternal mental health and children's emotional and behavioral development endures through childhood and adolescence; and b) whether this association is distinct for internalizing and externalizing symptoms.

## Methods

### Participants

The present study is embedded within the Rhea study, an ongoing population-based mother–child cohort in Crete, Greece. Over a 12-month period (February 2007-February 2008), 1,610 pregnant women agreed to participate with 1,363 singleton pregnancies followed up to delivery. Inclusion criteria required participants to reside in Heraklion, be over 16 years old, and understand Greek. Mothers were assessed during pregnancy, delivery, and postpartum, while children were evaluated at multiple stages: infancy (around 9 and 18 months), early childhood (around 4.2 years), mid-childhood (around 6.5 years), pre-adolescence (around 11 years), and adolescence (15 years). An in-depth evaluation of maternal and child mental health occurred at the 15-year follow-up as part of the IntExt Trajectories project. Further details on the cohort, follow-up visits, and measures can be found in Chatzi et al. [45].

**Fig. 1:**
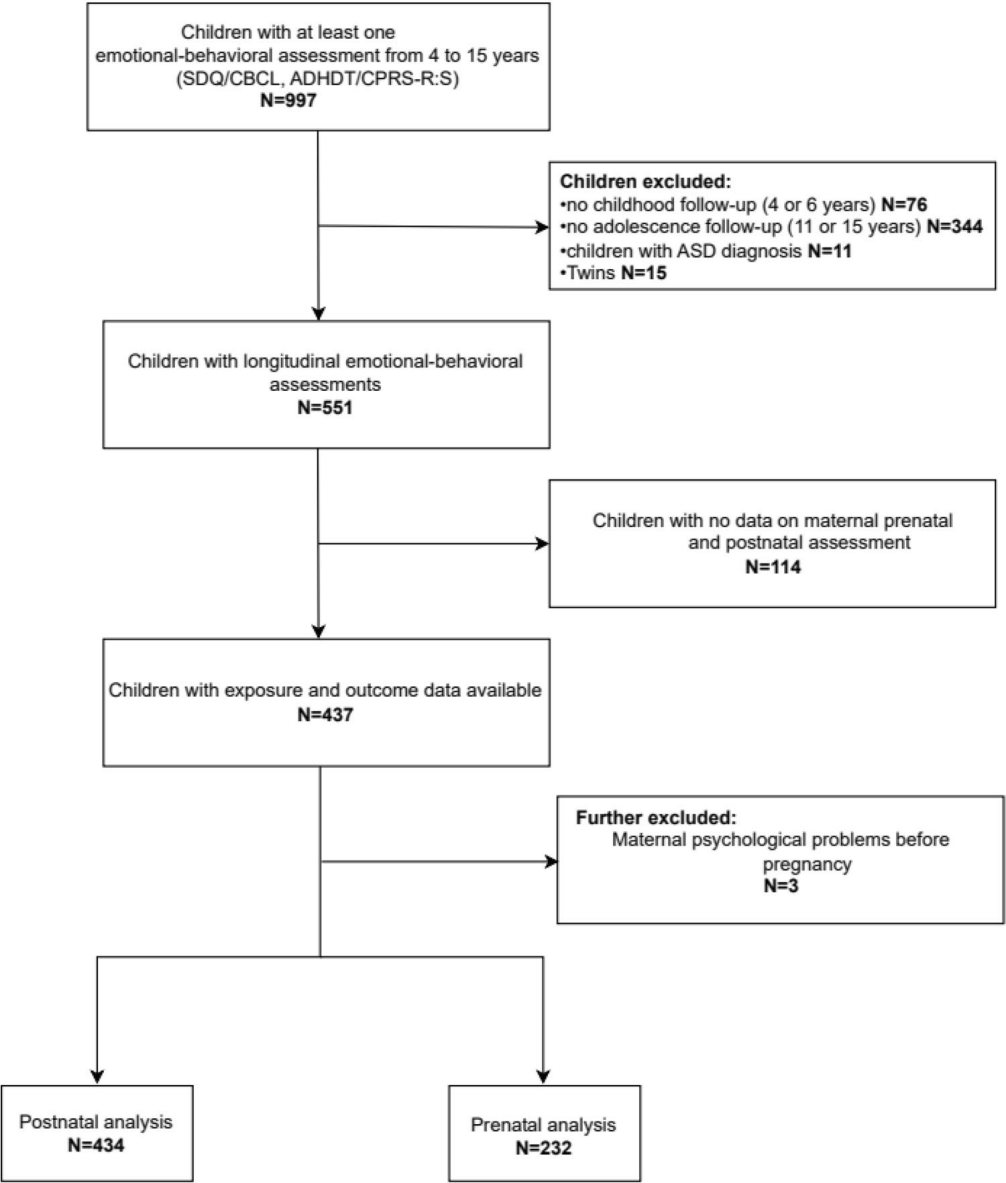
Flowchart of participants included in perinatal maternal mental health analyses. Abbreviations: ADHDT: Attention Deficit Hyperactivity Disorder Test; ASD: Autism Spectrum Disorder; CBCL: Child Behavior Checklist; CPRS-R:S: Conners’ Parent Rating Scale-Revised: Short Form; SDQ: Strengths and Difficulties Questionnaire

To assess emotional and behavioral outcomes over time, we analyzed follow-up data from 997 children assessed at ages 4, 6, 11, and 15. We required at least two assessments—one in childhood (ages 4 or 6) and one in adolescence (ages 11 or 15)—and excluded twins (*n* = 15) and children with an autism spectrum disorder (*n* = 11), yielding a final sample of 551 children. For maternal mental health data, we had information on 437 mothers postnatally and 232 antenatally. After excluding three mothers with a history of psychiatric disorders, the final sample included 434 mother–child pairs for postnatal and 232 for antenatal analyses (Fig. 1).

The present study was conducted according to the principles of the Helsinki Declaration, and all procedures and evaluations were approved by the Ethics Committee of the University Hospital of Heraklion (reference number: 96/06.02.2007) at baseline and by the Research Ethics Committee of the University of Crete (reference number: 43/16.03.2022) at the most recent follow-up (IntExt Trajectories project). Written informed consent was obtained from all participants.

### Measures

#### Maternal mental health assessment

Maternal depressive symptoms, trait anxiety, and personality characteristics were assessed at the third trimester of pregnancy (28–32 weeks). Postnatal depressive symptoms were assessed 8–10 weeks after childbirth.

Maternal depressive symptoms were measured using the Edinburgh Postnatal Depression Scale (EPDS) [46], a 10-item self-report tool rated on a Likert scale (0 to 3), with higher scores indicating more severe symptoms. The EPDS has strong psychometric properties for both antenatal and postnatal periods [47, 48] and has been validated in Greece [49, 50]. The EPDS score was analyzed both continuously and categorically in this study. A cut-off score of 13 was used to indicate high depressive symptoms consistent with prior research [46].

Maternal trait anxiety was assessed using the trait subscale of the State-Trait Anxiety Inventory (STAI) [51], which includes 20 items rated from 1 (“almost never”) to 4 (“almost always”). Higher scores indicate greater levels of trait anxiety. The STAI has been and validated in Greek [52].

Personality characteristics were evaluated using the Eysenck Personality Questionnaire-Revised (EPQ-R) [53], which assesses Neuroticism, Extraversion, and Psychoticism across 106 items. A Lie scale is included to identify socially desirable responses. The EPQ-R was culturally adapted for Greek speakers by the Rhea team, following a comprehensive translation process [54].

#### Children’s internalizing and externalizing difficulties

Mothers assessed their children's internalizing and externalizing symptoms using the Strengths and Difficulties Questionnaire (SDQ) [55] at age 4 and the Child Behavior Checklist (CBCL) [56] at ages 6, 11, and 15.

The SDQ is a 25-item tool that evaluates emotional and behavioral strengths and difficulties in children aged 4–17 years, adapted for the Greek population [57]. Items are rated on a 3-point Likert scale (0 to 2). The SDQ consists of five subscales and provides two broader scales: Internalizing (Emotional symptoms and Peer problems) and Externalizing (Conduct problems and Hyperactivity) difficulties, which were used in this study.

The CBCL is a widely used questionnaire consisting of 113 items that assesses adaptive and maladaptive functioning in children aged 6 to 18 years, also adapted for Greek contexts [58]. Items are rated on a 3-point Likert scale (0 to 2). The CBCL provides summaries of empirically-based syndrome scales and DSM-oriented scales, focusing on broad-band Internalizing (Anxiety/Depression, Withdrawal/Depression, and Somatic Complaints) and Externalizing (Rule-breaking Behavior and Aggressive Behavior) problems in this study.

#### Children’s ADHD symptoms

Mothers assessed their children’s ADHD symptoms using the Attention Deficit Hyperactivity Disorder Test (ADHDT) [59] at 4 years, and the Conners’ Parent Rating Scale-Revised: Short Form (CPRS-R: S) [60] at ages 6, 11, and 15.

The ADHDT comprises 36 items assessing ADHD-related symptoms based on DSM-IV criteria and has been adapted for the Greek population [61]. Items are rated on a 3-point Likert scale (0 to 2). It consists of three subscales: Hyperactivity, Inattention, and Impulsivity, along with a total ADHD difficulties used in this study, with higher scores reflecting greater severity and frequency of ADHD symptoms.

The CPRS-R: S is a 27-item questionnaire assessing ADHD symptoms over the past month using a Likert scale (0 to 3). It includes subscales for Oppositional problems, Cognitive problems/Inattention, and Hyperactivity, along with a total ADHD symptoms index used in this study, where higher scores indicate more severe symptoms. The CPRS-R: S was translated and adapted for the Greek population by the Rhea team, following a comprehensive translation process [54].

### Procedure

Women completed self-reported questionnaires for antenatal psychological assessment at 28–32 weeks of gestation, returning them by mail. For postnatal assessment at 8–10 weeks postpartum, trained interviewers conducted telephone interviews using the EPDS scale to evaluate symptoms of postnatal depression. Longitudinal evaluations of children’s behavioral and emotional development were performed by trained psychologists through validated questionnaires completed via parental reports. During follow-ups, mothers were informed about the study and invited to participate, with options for completing questionnaires either face-to-face or through a digital platform (i.e. at the most recent follow-up). Written consent was obtained from both mothers and their adolescent children at the most recent follow-up. After participation, mothers received a personalized psychological report on their children's development and could schedule a counseling session with Rhea team psychologists.

### Statistical analysis

Descriptive statistics were used to summarize the characteristics of the sample, the exposures and the outcomes of the study. Means with standard deviations (SD) and frequencies with percentages were calculated for continuous and categorical variables, respectively. Continuous variables were assessed for normality of the distribution by Shapiro–Wilk test and non-parametric tests of association have been performed when appropriate. Bivariate associations with the psychometric scales were examined using Mann–Whitney or Kruskal–Wallis for categorical characteristics and Spearman’s correlation coefficient between continuous variables.

To account for missing data total scores were prorated when less than 25% of items were missing across all analyzed scales. The distribution percentiles of the internalizing, externalizing and ADHD scales were calculated and used in all analyses, in order to harmonize the scales.

We examined associations between maternal mental health scales and emotional, behavioural and ADHD symptoms trajectories from 4 to 15 years of age by applying multivariate mixed regression models with random child intercept and a random slope for age of follow-up. In order to test potential time-varying effects, we included an interaction term between maternal mental health scales and child's age at follow-up. In order to evaluate the effect modification by sex, we introduced in each model a multiplicative interaction term between each exposure and the binary sex variable (male, female) and then, using the model with the multiplicative interaction term, we calculated the respective effect estimates for each sex. Estimates are reported as beta coefficients with their corresponding 95% confidence intervals (CI).

Potential confounders that were either associated with the outcomes or the exposure of interest in bivariate analyses with a *p*-value < 0.2 were included in the multivariable models, along with a priori selected confounders. We assessed collinearity of covariates in the models using the variance inflation factor (VIF < 10). Consequently, multivariate analyses were adjusted for child sex (male, female), exact age at assessment (years), maternal age at delivery (years), maternal smoking during pregnancy (ever, never), gestational age (completed weeks) or preterm birth (yes, no), breastfeeding duration (months), maternal and paternal educational level (low level: ≤ 9 years of mandatory schooling, medium level: > 9 years of mandatory schooling up to attending post-secondary school education, high level: attending university or having a university/technical college degree), birth order (first, second, third or more) and area of living (urban, rural) or maternal working status during pregnancy (paid job, not working/unemployed).

Finally, we performed sensitivity analyses in which we excluded preterm (< 37 gestational weeks) and low birth weight newborns (< 2500 g). In addition, mixed linear model analysis was performed excluding children with a diagnosis of learning disability or ADHD.

All hypothesis testing was conducted assuming a 0.05 significance level and a two-sided alternative hypothesis. All statistical analyses were conducted using Stata software, version 13.0 (Stata Corp, College Station, TX).

## Results

### Descriptive characteristics of study population

Parental and offspring characteristics of the sample are presented in Table 1. The mean maternal age at delivery was 30.0 years (± 4.6). Most mothers had attained medium (51.2%) or high (38.6%) educational level. The majority of women were employed during pregnancy (80.2%), lived in urban areas (78.6%) and were predominantly of Greek origin (94.9%). Among children, 53.7% were male and 46.3% were female, 52 (12.0%) were born preterm, nearly half of the children (46.2%) were the firstborn in their families and by the age of 15 years, 23 children (5.3%) had been diagnosed with learning disabilities and 12 children (2.8%) had an ADHD diagnosis.

**Table 1:** Parental and offspring characteristics of the sample (*N* = 434)

	*N*	% or mean (SD)		*N*	% or mean (SD)
Parental characteristics			Offspring characteristics		
Maternal age at delivery (years)	432	30.0 (4.6)	Sex		
Maternal education			Male	233	53.7
Low	44	10.2	Female	201	46.3
Medium	220	51.2	Gestational age (weeks)	432	38.2 (1.5)
High	166	38.6	Preterm birth		
Maternal working status during pregnancy			Yes	52	12.0
Employed	340	80.2	No	380	88.0
Not working/Unemployed	84	19.8	Birth anthropometry		
Marital status			Weight (kg)	433	3.2 (0.4)
Married	387	90.9	Length (cm)	429	50.6 (2.1)
Other	39	9.2	Head circumference (cm)	422	34.2 (1.3)
Paternal education			Birth order		
Low	127	30.0	First	200	46.2
Medium	185	43.7	Second	157	36.3
High	111	26.2	Third or more	76	17.6
Area of living			Breastfeeding		
Urban	341	78.6	Never	49	11.5
Rural	93	21.4	Ever	376	88.5
Family origin			Breastfeeding duration (months)	425	4.2 (4.1)
Greek	406	94.9	Nursery before 2 years		
Foreign/Mixed	22	5.1	Yes	101	23.3
Household income (tertiles)			No	332	76.7
Low (< 761.65 €/month)	64	20.9	Diagnosis		
Middle (775.27–1077.5 €/month)	100	32.6	None	399	91.9
High (1079.6–2179.3 €/month)	143	46.6	Learning disabilities	23	5.3
Smoking during pregnancy			ADHD	12	2.8
Never	265	62.5			
Ever	159	37.5			

Abbreviations: *ADHD* Attention Deficit Hyperactivity Disorder

Differences in socioeconomic status, birth characteristics, breastfeeding duration and nursery before two years were found between participants and non-participants (Supplementary Table 1). Participants had higher levels of education, were more likely to be employed during pregnancy and to have higher household income. Additionally, they had more favorable birth characteristics, longer durations of breastfeeding and they were more likely to enroll their children in nursery before the age of two years. Concerning mental health assessment, participants exhibited lower levels of extraversion and psychoticism compared to non-participants.

### Bivariate associations between perinatal maternal mental health and offspring internalizing, externalizing and ADHD symptoms per time point assessment

Table 2 illustrates the bivariate associations between perinatal maternal mental health and offspring outcomes at various ages. Maternal trait anxiety was consistently linked to higher internalizing symptoms at every assessment, externalizing symptoms from ages 4 to 11, and ADHD symptoms at ages 4 and 6. Among maternal personality traits, neuroticism was the only one significantly associated with child outcomes, showing correlations with higher internalizing and externalizing symptoms across all ages and increased ADHD symptoms from ages 4 to 11. Maternal antenatal depressive symptoms were positively associated with children's emotional symptoms from ages 4 to 15, behavioral symptoms from ages 4 to 11, and ADHD symptoms at ages 4 and 6. Higher postnatal depressive symptoms were significantly correlated with increased internalizing and ADHD symptoms from ages 4 to 11 and with externalizing symptoms at all ages.

**Table 2:** Correlations between perinatal maternal mental health and internalizing, externalizing, and ADHD symptoms

	4 years		6 years		11 years		15 years	
	*N*	rho	*N*	rho	*N*	rho	*N*	rho
**Internalizing symptoms**								
STAI-Trait anxiety	214	0.196**	194	0.234***	149	0.221**	203	0.167*
EPQ-Psychoticism	206	0.124	186	0.113	141	0.005	193	0.091
EPQ-Extraversion	206	−0.050	186	−0.010	141	−0.053	193	−0.028
EPQ-Neuroticism	205	0.208**	186	0.209**	141	0.316***	192	0.200**
EPDS Antenatal	205	0.194**	186	0.158*	141	0.260**	195	0.142*
EPDS Postnatal	407	0.192***	347	0.219***	259	0.233***	375	0.034
**Externalizing symptoms**								
STAI-Trait anxiety	213	0.237***	195	0.233***	149	0.241**	202	0.104
EPQ-Psychoticism	205	0.101	187	−0.029	141	−0.121	192	0.059
EPQ-Extraversion	205	0.022	187	−0.120	141	−0.067	192	0.064
EPQ-Neuroticism	204	0.282***	187	0.267***	141	0.337***	191	0.189**
EPDS Antenatal	204	0.194**	187	0.214**	141	0.240**	194	0.131
EPDS Postnatal	406	0.212***	349	0.290***	259	0.229***	374	0.103*
**ADHD symptoms**								
STAI-Trait anxiety	213	0.176**	193	0.215**	149	0.122	202	0.032
EPQ-Psychoticism	205	0.129	185	0.054	142	0.016	192	0.085
EPQ-Extraversion	205	0.125	185	−0.088	142	0.054	192	0.093
EPQ-Neuroticism	204	0.190**	185	0.191**	142	0.190*	191	0.111
EPDS Antenatal	204	0.180**	185	0.160*	142	0.091	194	0.014
EPDS Postnatal	406	0.183***	344	0.193***	261	0.162**	376	0.050

Abbreviations: *ADHD* Attention Deficit Hyperactivity Disorder; *EPDS* Edinburgh Postnatal Depression Scale; *EPQ* Eysenck Personality Questionnaire; *STAI* State-Trait Anxiety Inventory

* *p* < 0.05. ** *p* < 0.01. *** *p* < 0.001

### Multivariate associations between perinatal maternal mental health and trajectories of internalizing, externalizing and ADHD symptoms from 4 to 15 years of age, mixed model analyses

The results from mixed model analyses regarding aspects of maternal mental health and offspring’s outcomes from 4 to 15 years are presented in Table 3. Maternal trait anxiety and neuroticism were associated with increased internalizing symptoms across development (b = 0.68, 95%CI: 0.39, 0.98 and b = 1.57, 95%CI: 1.06, 2.08, respectively). Increased maternal antenatal depressive symptoms (examined both as continuous and dichotomous variable) were positively associated with offspring’s emotional symptoms from early childhood through adolescence (b = 1.22, 95%CI: 0.69, 1.75 and b = 15.99, 95%CI: 9.33, 22.66, respectively). Similarly, maternal postnatal depressive symptoms were linked to increased emotional difficulties across ages (b = 0.88, 95%CI: 0.44, 1.32 for continuous variable and b = 6.98, 95%CI: 0.19, 13.77 for dichotomous variable).

**Table 3:** Adjusted associations of maternal mental health and trajectories of internalizing, externalizing and ADHD symptoms from 4 to 15 years of age, mixed model analyses

	Across ages		
	*N*	b (95% CI)	*p*-value
**Internalizing symptoms** ^a^			
STAI-Trait anxiety	222	0.68 (0.39, 0.98)	** < 0.001**
EPQ-Psychoticism	211	0.89 (−0.29, 2.08)	0.139
EPQ-Extraversion	211	−0.51 (−1.17, 0.15)	0.129
EPQ-Neuroticism	210	1.57 (1.06, 2.08)	** < 0.001**
EPDS Antenatal	213	1.22 (0.69, 1.75)	** < 0.001**
EPDS Antenatal (≥ 13)	213	15.99 (9.33, 22.66)	** < 0.001**
EPDS Postnatal	406	0.88 (0.44, 1.32)	** < 0.001**
EPDS Postnatal (≥ 13)	406	6.98 (0.19, 13.77)	**0.044**
**Externalizing symptoms** ^b^			
STAI-Trait anxiety	221	0.64 (0.29, 0.98)	** < 0.001**
EPQ-Psychoticism	210	−0.00 (−1.39, 1.38)	0.997
EPQ-Extraversion	210	−0.40 (−1.08, 0.27)	0.239
EPQ-Neuroticism	209	1.60 (1.06, 2.13)	** < 0.001**
EPDS Antenatal	212	0.92 (0.33, 1.50)	**0.002**
EPDS Antenatal (≥ 13)	212	6.12 (−1.65, 13.88)	0.123
EPDS Postnatal	404	1.11 (0.67, 1.55)	** < 0.001**
EPDS Postnatal (≥ 13)	404	9.65 (3.32, 15.98)	**0.003**
**ADHD symptoms** ^b^			
STAI-Trait anxiety	221	0.39 (0.07, 0.70)	**0.016**
EPQ-Psychoticism	211	0.32 (−0.91, 1.55)	0.611
EPQ-Extraversion	211	0.05 (−0.61, 0.70)	0.887
EPQ-Neuroticism	210	0.95 (0.42, 1.47)	** < 0.001**
EPDS Antenatal	213	0.48 (−0.08, 1.04)	0.095
EPDS Antenatal (≥ 13)	213	3.20 (−4.25, 10.65)	0.400
EPDS Postnatal	406	0.74 (0.30, 1.18)	**0.001**
EPDS Postnatal (≥ 13)	406	8.37 (2.09, 14.64)	**0.009**

Abbreviations: *ADHD* Attention Deficit Hyperactivity Disorder; *EPDS* Edinburgh Postnatal Depression Scale; *EPQ* Eysenck Personality Questionnaire; *STAI* State-Trait Anxiety Inventory

^a^ Adjusted for child sex and exact age at assessment, maternal age, maternal smoking during pregnancy, preterm birth, breastfeeding duration, maternal education, paternal education, birth order and urban area of living

^b^ Adjusted for child sex and exact age at assessment, maternal age, maternal smoking during pregnancy, gestational age, breastfeeding duration, maternal education, paternal education, birth order and maternal working status

Bold font indicates *p* < 0.05

Higher levels of maternal trait anxiety and neuroticism were linked to elevated behavioral symptoms across ages (b = 0.64, 95%CI: 0.29, 0.98 and b = 1.60, 95%CI: 1.06, 2.13, respectively). Antenatal maternal depressive symptoms were associated with increased externalizing problems (b = 0.92, 95%CI: 0.33, 1.50). In addition, higher levels of maternal depressive symptoms at the postnatal period were related to more behavioral difficulties in children from 4 to 15 years of age (b = 1.11, 95%CI: 0.67, 1.55 for continuous variable and b = 9.65, 95%CI: 3.32, 15.98 for dichotomous variable).

Similarly with internalizing and externalizing symptoms, maternal trait anxiety and the personality trait of neuroticism were linked to increased ADHD symptoms from 4 to 15 years (b = 0.39, 95%CI: 0.07, 0.70 and b = 0.95, 95%CI: 0.42, 1.47, respectively). Postnatal, but not antenatal, maternal depressive symptoms were associated with higher symptoms of ADHD across child development (b = 0.74, 95%CI: 0.30, 1.18 for continuous variable and b = 8.37, 95%CI: 2.09, 14.64 for dichotomous variable).

### Interaction effect of age and sex

**Fig. 2:**
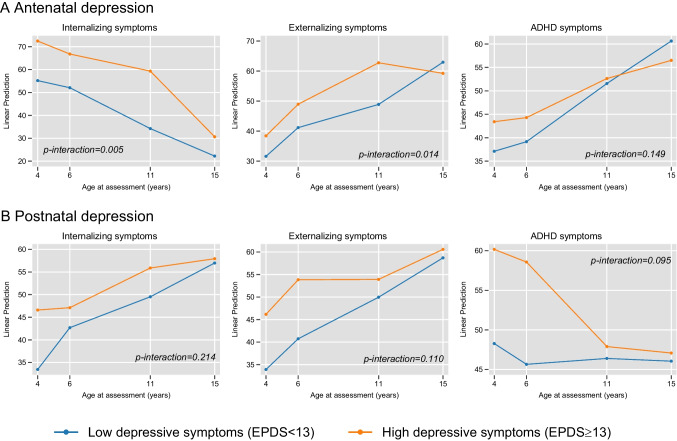
Age interaction and adjusted associations of maternal depression (as dichotomous variable) and trajectories of internalizing, externalizing and ADHD symptoms from ages 4 to 15 years, mixed model analyses.*Abbreviations: *ADHD: Attention Deficit Hyperactivity Disorder; EPDS: Edinburgh Postnatal Depression Scale

To assess variations over time, mixed models included interactions between maternal mental health exposures and child age at follow-up (Table 4 and Fig. 2). Significant interactions for internalizing symptoms showed that children of mothers with high antenatal depression (dichotomous variable) had elevated symptoms at ages 4, 6, and 11, but not at 15. A significant age interaction showed that postnatal depression was linked to increased emotional symptoms from ages 4 to 11, with no effect at 15. Antenatal depression (dichotomous variable) was not associated with externalizing symptoms overall, but elevated symptoms were seen at age 11. Postnatal depression showed increased behavioral difficulties at ages 4, 6, and 11, but not at 15, and maternal extraversion was linked to decreased ADHD symptoms at age 6.

**Table 4:** Age interaction and adjusted associations of perinatal maternal mental health and trajectories of internalizing, externalizing and ADHD symptoms from ages 4 to 15 years, mixed model analyses

		4 years		6 years		11 years		15 years		*p* interaction
	*N*	b (95% CI)	*p*-value	b (95% CI)	*p*-value	b (95% CI)	*p*-value	b (95% CI)	*p*-value	with age
**Internalizing symptoms** ^a^										
STAI-Trait anxiety	222	0.66 (0.24, 1.07)	**0.002**	0.79 (0.38, 1.20)	** < 0.001**	0.77 (0.35, 1.20)	** < 0.001**	0.52 (0.09, 0.96)	**0.019**	0.567
EPQ-Psychoticism	211	1.36 (−0.25, 2.96)	0.099	0.67 (−1.05, 2.40)	0.443	0.14 (−1.70, 1.98)	0.879	1.02 (−0.60, 2.64)	0.218	0.687
EPQ-Extraversion	211	−0.76 (−1.75, 0.23)	0.130	−0.10 (−1.13, 0.93)	0.855	−0.54 (−1.57, 0.49)	0.305	−0.60 (−1.55, 0.34)	0.211	0.718
EPQ-Neuroticism	210	1.37 (0.62, 2.13)	** < 0.001**	1.60 (0.86, 2.33)	** < 0.001**	2.27 (1.49, 3.05)	** < 0.001**	1.29 (0.55, 2.03)	**0.001**	0.173
EPDS Antenatal	213	1.25 (0.53, 1.97)	**0.001**	1.16 (0.39, 1.93)	**0.003**	1.73 (0.97, 2.48)	** < 0.001**	0.88 (0.14, 1.63)	**0.021**	0.275
EPDS Postnatal	406	1.09 (0.53, 1.65)	** < 0.001**	1.12 (0.47, 1.76)	**0.001**	1.21 (0.51, 1.90)	**0.001**	0.07 (−0.61, 0.74)	0.847	**0.011**
**Externalizing symptoms** ^b^										
STAI-Trait anxiety	221	0.66 (0.23, 1.08)	**0.002**	0.72 (0.30, 1.14)	**0.001**	0.76 (0.28, 1.24)	**0.002**	0.34 (−0.14, 0.82)	0.168	0.328
EPQ-Psychoticism	210	1.12 (−0.55, 2.80)	0.188	−0.73 (−2.37, 0.91)	0.384	−1.49 (−3.51, 0.53)	0.148	0.10 (−1.90, 2.10)	0.922	**0.047**
EPQ-Extraversion	210	−0.22 (−1.07, 0.63)	0.616	−0.88 (−1.80, 0.04)	0.061	−0.43 (−1.47, 0.61)	0.416	0.00 (−0.97, 0.97)	0.998	0.400
EPQ-Neuroticism	209	1.59 (0.92, 2.25)	** < 0.001**	1.54 (0.76, 2.32)	** < 0.001**	2.13 (1.29, 2.98)	** < 0.001**	1.29 (0.49, 2.08)	**0.002**	0.318
EPDS Antenatal	212	0.89 (0.18, 1.59)	**0.013**	1.17 (0.43, 1.91)	**0.002**	1.20 (0.38, 2.02)	**0.004**	0.42 (−0.44, 1.29)	0.334	0.236
EPDS Postnatal	404	1.16 (0.63, 1.68)	** < 0.001**	1.54 (0.95, 2.14)	** < 0.001**	0.95 (0.22, 1.69)	**0.011**	0.36 (−0.31, 1.03)	0.296	**0.009**
**ADHD symptoms** ^b^										
STAI-Trait anxiety	221	0.52 (0.09, 0.95)	**0.017**	0.52 (0.13, 0.90)	**0.009**	0.23 (−0.26, 0.72)	0.357	0.03 (−0.42, 0.48)	0.897	0.172
EPQ-Psychoticism	211	0.72 (−0.87, 2.32)	0.375	0.14 (−1.46, 1.75)	0.861	0.02 (−1.92, 1.96)	0.986	0.08 (−1.72, 1.89)	0.927	0.904
EPQ-Extraversion	211	0.58 (−0.35, 1.51)	0.219	−0.85 (−1.68, −0.03)	**0.043**	0.17 (−0.82, 1.17)	0.734	0.26 (−0.69, 1.21)	0.594	**0.008**
EPQ-Neuroticism	210	1.10 (0.37, 1.84)	**0.003**	1.01 (0.32, 1.69)	**0.004**	0.87 (0.05, 1.70)	**0.038**	0.62 (−0.20, 1.45)	0.138	0.799
EPDS Antenatal	213	0.85 (0.13, 1.57)	**0.021**	0.64 (−0.05, 1.33)	0.071	0.13 (−0.66, 0.93)	0.743	−0.22 (−1.06, 0.61)	0.600	0.061
EPDS Postnatal	406	0.98 (0.41, 1.55)	**0.001**	0.97 (0.31, 1.63)	**0.004**	0.38 (−0.35, 1.10)	0.307	0.28 (−0.38, 0.94)	0.403	0.181

Abbreviations: *ADHD* Attention Deficit Hyperactivity Disorder; *EPDS* Edinburgh Postnatal Depression Scale; *EPQ* Eysenck Personality Questionnaire; *STAI* State-Trait Anxiety Inventory

^a^ Adjusted for child sex and exact age at assessment, maternal age, maternal smoking during pregnancy, preterm birth, breastfeeding duration, maternal education, paternal education, birth order and urban area of living

^b^ Adjusted for child sex and exact age at assessment, maternal age, maternal smoking during pregnancy, gestational age, breastfeeding duration, maternal education, paternal education, birth order and maternal working status

Bold font indicates *p* < 0.05

Sex interactions were examined in separate mixed models, revealing no differences by child sex in the relationship between perinatal maternal mental health and internalizing, externalizing and ADHD symptoms (Supplementary Table 2).

### Further sensitivity analyses

We conducted several sensitivity analyses by excluding preterm newborns and/or low birth weight offspring (Supplementary Table 3), finding no substantial changes in the results. Similarly, removing children diagnosed with learning disabilities or ADHD diagnosis (Supplementary Table 4) did not significantly affect the observed associations.

## Discussion

The present study indicated long-term associations between perinatal maternal mental health and children's emotional and behavioral outcomes from ages 4 to 15. Specifically, elevated levels of maternal trait anxiety and neuroticism were linked to increased internalizing, externalizing, and ADHD symptoms in children. Both antenatal and postnatal maternal depressive symptoms were associated with a higher incidence of emotional and behavioral problems over time, but only postnatal depression was related to an increase in ADHD symptoms.

To our knowledge, this is the first study to investigate the role of maternal personality along with various aspects of perinatal mental health in the emotional and behavioral symptom trajectories of offspring. Our findings indicate that maternal neuroticism is longitudinally linked to increased internalizing, externalizing, and ADHD symptoms in children. Neuroticism, characterized by emotional instability, stress susceptibility, and vulnerability [36, 62], may predispose offspring to emotional and behavioral issues, given that up to 80% of neuroticism differences are hereditary [63]. Additionally, high neuroticism may influence child development through its associations with psychosocial factors, such as suboptimal parenting, which are known to affect the severity of internalizing, externalizing, and ADHD symptoms [64, 65].

In our study, both maternal antenatal and postnatal depressive symptoms were associated with higher offspring internalizing and externalizing symptoms, while postpartum depression was specifically linked to ADHD symptomatology from early childhood through adolescence. Our findings agree with studies showing that maternal postpartum depression is associated with the internalizing symptom trajectories from toddlerhood to middle childhood [23, 24], as well as with the internalizing and externalizing symptom trajectories up to adolescence [25]. While previous studies link antenatal depression to children’s internalizing, externalizing, and ADHD symptoms across developmental stages [12, 14, 20], current literature indicates that it predicts emotional and behavioral problem trajectories from toddlerhood to middle childhood primarily in conjunction with anxiety [34]. Our findings contrast with Kallas et al. [34], showing that maternal depression's lasting impact on emotional and behavioral development begins during pregnancy.

Furthermore, we found that higher levels of maternal antenatal anxiety were associated with increased internalizing, externalizing, and ADHD symptoms from early childhood to adolescence. This aligns with O'Donnell et al. [35], who found similar effects in the ALSPAC cohort, where maternal antenatal anxiety impacted emotional and behavioral problems throughout development. In contrast, Kallas et al. [34] did not find significant links between antenatal anxiety and symptom trajectories in the French EDEN cohort, since only comorbid maternal anxiety and depression were linked to children’s adverse outcomes children. The differences in findings may be attributed to several factors, including cultural variations in stressors and support systems during pregnancy, differences in mental health risk and protective factors affecting the child's development, and variations in analytical methods used.

The mechanisms through which antenatal and postnatal distress influence child mental health outcomes vary [6]. Antenatal depression and anxiety impact offspring development mainly through biological pathways, such as DNA methylation [66], altered brain connectivity and epigenetic changes [67–69], while postpartum depression affects emotional and behavioral outcomes through psychosocial factors like parental stress, maternal bonding, and co-parenting support [9, 18, 70, 71].

In our study, perinatal maternal mental health showed age-specific associations with offspring emotional and behavioral development, with stronger effects in early and middle childhood that became insignificant during adolescence. Specifically, maternal antenatal and postpartum depression were linked to increased internalizing and externalizing symptoms at ages 4, 6, and 11, but not at age 15, supporting the idea that the impact of maternal mental health diminishes over time [72]. This finding aligns with the notion that during adolescence, factors such as peer relationships may moderate maternal mental health's influence [73]. One possible explanation for this finding may be the increasing influence of peer relationships during adolescence, which become central to adolescents'social and emotional development. These relationships, along with the emergence of romantic connections, may provide adolescents with important emotional support and mechanisms for self-regulation, thereby moderating the effects of maternal mental health on their well-being [73]. Additionally, in the context of the digital age, adolescents increasingly rely on online communication to maintain and strengthen offline friendships. These digital interactions, which facilitate the arrangement of meet-ups, the development of intimacy, and expressions of affection, have been associated with enhanced well-being [74]. Thus, such online interactions with peers may serve as protective factors that help buffer the negative effects of maternal mental health difficulties, potentially contributing to the observed attenuation of its impact during adolescence.

Notably, while both antenatal and postpartum depression affected development, antenatal depression had a stronger impact at age 11, whereas postpartum depression was more influential at ages 4 and 6. This may reflect different mechanisms: antenatal depression could create latent vulnerabilities through biological pathways [11, 67, 68], while postpartum depression may disrupt mother-infant bonding and caregiving abilities [9, 18, 70], which are crucial for early development [75].

### Strengths and limitations

A key strength of this study is the longitudinal assessment of children's internalizing, externalizing, and ADHD symptoms from early childhood through adolescence, using reliable and comprehensive psychometric measures. Also, we considered a wide range of maternal mental health aspects capturing pre and postnatal period. Additionally, we adjusted for several important confounding variables by leveraging data from a well-established mother–child cohort.

Despite its strengths, our study has several limitations. Although we used psychometric tools with strong validity and reliability, the questionnaires for assessing outcomes at 4 years (SDQ, ADHDT) differed from those used at later ages (CBCL, CPRS-R:S). While these tools are correlated and measure similar constructs [76], the process of data harmonization, despite being applied with established methods, may have influenced our results. Another limitation is that, at the most recent follow-up, mothers had the option to complete the questionnaires either face-to-face or via a digital platform, which may have influenced how children’s emotional and behavioral difficulties were evaluated. However, only a small number of mothers (*n* = 5) chose the face-to-face option, and these responses were excluded from the final analysis due to other factors, such as the lack of prenatal and postnatal assessments. As a result, the final dataset comprised exclusively of digital completions. Furthermore, this study was conducted on a subsample of the population with available data, which may result in limited statistical power and/or selection bias. However, when comparing participants to non-participants we found only minor differences in socioeconomic status, birth characteristics, breastfeeding duration, nursery initiation and traits such as extraversion and psychoticism, which are not expected to significantly affect the qualitative conclusions regarding direction and approximate magnitude [77]. Additionally, although we adjusted for various confounding variables, residual confounding from unmeasured factors, such as current maternal mental health, could still be present. Moreover, the study did not account for key social and relational factors, including dyadic coping with stress, perceived social support, and family functioning, which may play a protective or moderating role in the relationship between maternal mental health and child developmental outcomes. The absence of these variables limits our ability to fully explain why the impact of perinatal maternal mental health appeared stronger in early and middle childhood than during adolescence.

## Conclusion

In conclusion, this study revealed significant associations between perinatal maternal mental health and trajectories of internalizing, externalizing, and ADHD difficulties from early childhood through adolescence. These symptoms are strongly linked with mental health and risky behaviors in adulthood. The findings underscore the importance of addressing maternal mental health to promote better long-term outcomes for children. The findings should be interpreted within the context of the socio-economic challenges faced by Greece, particularly the 2008 financial crisis and the COVID-19 pandemic, which likely exacerbated emotional and behavioral difficulties in children. The cumulative impact of these socio-economic stressors may have significantly intensified the developmental challenges observed throughout the course of the study. An integrated approach to maternal and child mental health, including early screening and tailored interventions for anxiety and depression, is essential. Future research should investigate the genetic and environmental mechanisms underlying this relationship, expand studies to include diverse populations, and enhance clinical practice by integrating maternal and child mental health care.

## Supplementary Information

Below is the link to the electronic supplementary material.

Supplementary file1 (DOCX 58 KB)

## Data Availability

No datasets were generated or analysed during the current study.
