# Supplementary material for: Perinatal maternal mental health and offspring internalizing and externalizing difficulties from early childhood through adolescence: Rhea mother—child cohort in Crete, Greece
Source: Eur Child Adolesc Psychiatry. 2025 May 21;34(10):3261–74. doi: 10.1007/s00787-025-02746-1 (PMC12592253; doi:10.1007/s00787-025-02746-1)
Supplement: Supplementary file 1 — Supplementary file1 (DOCX 58 KB) [file 787_2025_2746_MOESM1_ESM.docx]

**Supplementary Table 1.** Parental and offspring characteristics of the sample included (n=434) compared to those excluded from analyses (n=563).

|  | **Participants** | | **Non participants** | |  |
| --- | --- | --- | --- | --- | --- |
|  | **N** | **% or Mean (SD)** | **N** | **% or Mean (SD)** | **p-value** |
| **Parental characteristics** |  |  |  |  |  |
| **Maternal age at delivery** (years) | 432 | 30.0 (4.6) | 557 | 29.9 (5.2) | 0.770 |
| **Maternal education** |  |  |  |  |  |
| Low | 44 | 10.2 | 107 | 20.2 | <0.001 |
| Medium | 220 | 51.2 | 275 | 51.8 |  |
| High | 166 | 38.6 | 149 | 28.1 |  |
| **Maternal working status during pregnancy** |  |  |  |  |  |
| Employed | 84 | 19.8 | 141 | 27.8 | 0.005 |
| Not working/unemployed | 340 | 80.2 | 367 | 72.2 |  |
| **Maternal marital status** |  |  |  |  |  |
| Married | 387 | 90.8 | 451 | 86.9 | 0.057 |
| Other | 39 | 9.2 | 68 | 13.1 |  |
| **Paternal education** |  |  |  |  |  |
| Low | 127 | 30.0 | 183 | 35.8 | 0.161 |
| Medium | 185 | 43.7 | 210 | 51.1 |  |
| High | 111 | 26.2 | 118 | 23.1 |  |
| **Area of living** |  |  |  |  |  |
| Urban | 341 | 78.6 | 320 | 74.6 | 0.167 |
| Rural | 93 | 21.4 | 109 | 25.4 |  |
| **Family origin** |  |  |  |  |  |
| Both Greek | 406 | 94.9 | 476 | 92.1 | 0.065 |
| Both foreign | 5 | 1.2 | 18 | 3.5 |  |
| Mixed | 17 | 4.0 | 23 | 4.4 |  |
| **Household income** (tertiles) |  |  |  |  |  |
| Low | 96 | 25.6 | 162 | 40.6 | <0.001 |
| Middle | 134 | 35.7 | 124 | 31.1 |  |
| High | 145 | 38.7 | 113 | 28.3 |  |
| **Offspring characteristics** |  |  |  |  |  |
| **Sex** |  |  |  |  |  |
| Male | 233 | 53.7 | 291 | 51.7 | 0.531 |
| Female | 201 | 46.3 | 272 | 48.3 |  |
| **Gestational** **age** (weeks) | 432 | 38.2 (1.5) | 531 | 38.0 (1.8) | 0.026 |
| **Preterm birth** |  |  |  |  |  |
| Yes | 52 | 12.0 | 90 | 16.9 | 0.032 |
| No | 380 | 88.0 | 441 | 83.1 |  |
| **Birth anthropometry** |  |  |  |  |  |
| Weight (kg) | 433 | 3.2 (0.4) | 523 | 3.1 (0.5) | <0.001 |
| Length (cm) | 433 | 50.6 (2.1) | 544 | 49.9 (2.8) | <0.001 |
| Head circumference (cm) | 433 | 34.2 (1.3) | 544 | 33.9 (1.8) | 0.007 |
| **Intensive care unit** |  |  |  |  |  |
| Yes | 74 | 18.2 | 92 | 19.5 | 0.633 |
| No | 332 | 81.8 | 380 | 80.5 |  |
| **Birth order** |  |  |  |  |  |
| First | 200 | 46.2 | 198 | 44.9 | 0.922 |
| Second | 157 | 36.3 | 165 | 37.4 |  |
| Third or more | 76 | 17.6 | 78 | 17.7 |  |
| **Breastfeeding** |  |  |  |  |  |
| Never | 49 | 11.5 | 89 | 17.5 | 0.011 |
| Ever | 376 | 88.5 | 420 | 82.5 |  |
| **Breastfeeding duration** (months) | 425 | 4.2 (4.1) | 509 | 3.6 (4.2) | 0.023 |
| **Nursery before 2 years** |  |  |  |  |  |
| No | 332 | 76.7 | 433 | 82.0 | 0.041 |
| Yes | 101 | 23.3 | 95 | 18.0 |  |
| **Maternal mental health** |  |  |  |  |  |
| STAI-Trait anxiety | 228 | 39.8 (8.9) | 218 | 38.8 (8.6) | 0.225 |
| EPQ-Psychoticism | 218 | 6.3 (2.4) | 211 | 6.8 (2.6) | 0.044 |
| EPQ-Extraversion | 218 | 14.1 (4.2) | 211 | 15.3 (3.7) | 0.003 |
| EPQ-Neuroticism | 219 | 13.2 (4.9) | 211 | 13.5 (4.7) | 0.592 |
| EPDS Antenatal | 219 | 7.7 (5.2) | 215 | 7.7 (4.8) | 0.941 |
| EPDS Antenatal (≥13) | 40 | 18.3 | 34 | 15.8 | 0.497 |
| EPDS Postnatal | 434 | 6.4 (4.9) | 334 | 6.6 (5.1) | 0.453 |
| EPDS Postnatal (≥13) | 54 | 12.4 | 43 | 12.9 | 0.858 |

*Abbreviations*: EPDS: Edinburgh Postnatal Depression Scale; EPQ: Eysenck Personality Questionnaire; STAI: State-Trait Anxiety Inventory.

**Supplementary Table 2.** Sex interaction and adjusted associations of perinatal maternal mental health and trajectories of internalizing, externalizing and ADHD symptoms from ages 4 to 15 years, mixed model analyses.

|  |  | **Males** | |  | **Females** | | **p interaction** |
| --- | --- | --- | --- | --- | --- | --- | --- |
|  | **N** | **b (95% CI)** | **p-value** |  | **b (95% CI)** | **p-value** | **with sex** |
| **Internalizing symptoms ^a^** |  |  |  |  |  |  |  |
| STAI-Trait anxiety | 222 | **0.70 (0.29, 1.12)** | **0.001** |  | **0.66 (0.26, 1.06)** | **0.001** | 0.887 |
| EPQ-Psychoticism | 211 | 1.04 (-0.47, 2.54) | 0.179 |  | 0.72 (-1.11, 2.55) | 0.441 | 0.792 |
| EPQ-Extraversion | 211 | -0.66 (-1.52, 0.20) | 0.130 |  | -0.31 (-1.32, 0.70) | 0.547 | 0.602 |
| EPQ-Neuroticism | 210 | **1.76 (1.02, 2.50)** | **<0.001** |  | **1.34 (0.65, 2.03)** | **<0.001** | 0.421 |
| EPDS Antenatal | 213 | **1.26 (0.51, 2.02)** | **0.001** |  | **1.16 (0.44, 1.89)** | **0.002** | 0.851 |
| EPDS Antenatal (≥13) | 213 | **17.86 (8.93, 26.79)** | **<0.001** |  | **11.85 (2.79, 20.92)** | **0.010** | 0.368 |
| EPDS Postnatal | 406 | **0.90 (0.27, 1.53)** | **0.005** |  | **0.86 (0.27, 1.45)** | **0.004** | 0.931 |
| EPDS Postnatal (≥13) | 406 | 6.57 (-2.26, 15.41) | 0.145 |  | 7.64 (-2.61, 17.90) | 0.144 | 0.875 |
| **Externalizing symptoms ^b^** |  |  |  |  |  |  |  |
| STAI-Trait anxiety | 221 | **0.50 (0.03, 0.98)** | **0.038** |  | **0.84 (0.37, 1.31)** | **<0.001** | 0.324 |
| EPQ-Psychoticism | 210 | 0.29 (-1.53, 2.10) | 0.758 |  | -0.37 (-2.33, 1.58) | 0.708 | 0.616 |
| EPQ-Extraversion | 210 | -0.29 (-1.15, 0.57) | 0.512 |  | -0.56 (-1.57, 0.46) | 0.284 | 0.688 |
| EPQ-Neuroticism | 209 | **1.56 (0.76, 2.36)** | **<0.001** |  | **1.64 (0.93, 2.35)** | **<0.001** | 0.881 |
| EPDS Antenatal | 212 | 0.68 (-0.11, 1.46) | 0.090 |  | **1.24 (0.37, 2.10)** | **0.005** | 0.345 |
| EPDS Antenatal (≥13) | 212 | 4.93 (-3.99, 13.85) | 0.279 |  | 8.70 (-6.41, 23.81) | 0.259 | 0.674 |
| EPDS Postnatal | 404 | **1.08 (0.49, 1.67)** | **<0.001** |  | **1.15 (0.50, 1.80)** | **0.001** | 0.877 |
| EPDS Postnatal (≥13) | 404 | **8.16 (0.43, 15.90)** | **0.039** |  | **12.04 (1.46, 22.62)** | **0.026** | 0.558 |
| **ADHD symptoms ^b^** |  |  |  |  |  |  |  |
| STAI-Trait anxiety | 221 | 0.37 (-0.04, 0.79) | 0.080 |  | 0.41 (-0.04, 0.86) | 0.071 | 0.901 |
| EPQ-Psychoticism | 211 | -0.23 (-1.96, 1.51) | 0.800 |  | 1.04 (-0.56, 2.64) | 0.202 | 0.290 |
| EPQ-Extraversion | 211 | -0.11 (-0.97, 0.74) | 0.793 |  | 0.26 (-0.68, 1.20) | 0.585 | 0.549 |
| EPQ-Neuroticism | 210 | **1.12 (0.40, 1.85)** | **0.002** |  | **0.74 (0.03, 1.45)** | **0.042** | 0.446 |
| EPDS Antenatal | 213 | 0.53 (-0.16, 1.22) | 0.132 |  | 0.41 (-0.48, 1.29) | 0.366 | 0.827 |
| EPDS Antenatal (≥13) | 213 | 3.91 (-4.53, 12.36) | 0.364 |  | 1.62 (-12.91, 16.16) | 0.827 | 0.788 |
| EPDS Postnatal | 406 | **0.57 (0.00, 1.15)** | **0.050** |  | **0.95 (0.25, 1.64)** | **0.007** | 0.419 |
| EPDS Postnatal (≥13) | 406 | 6.51 (-0.95, 13.97) | 0.087 |  | **11.36 (0.48, 22.23)** | **0.041** | 0.467 |

*Abbreviations*: ADHD: Attention Deficit Hyperactivity Disorder; EPDS: Edinburgh Postnatal Depression Scale; EPQ: Eysenck Personality Questionnaire; STAI: State-Trait Anxiety Inventory.

**^a^** Adjusted for child sex and exact age at assessment, maternal age, maternal smoking during pregnancy, preterm birth, breastfeeding duration, maternal education, paternal education, birth order and urban area of living.

**^b^** Adjusted for child sex and exact age at assessment, maternal age, maternal smoking during pregnancy, gestational age, breastfeeding duration, maternal education, paternal education, birth order and maternal working status.

Bold font indicates p < 0.05.

**Supplementary Table 3.** Age interaction and adjusted associations of perinatal maternal mental health and trajectories of internalizing, externalizing and ADHD symptoms from ages 4 to 15 years, mixed model analyses **after excluding children born preterm and/or with low birth weight**.

|  |  |  | **Across ages** | **4 years** | **6 years** | **11 years** | **15 years** | **p interaction** |
| --- | --- | --- | --- | --- | --- | --- | --- | --- |
|  | **N** |  | **b (95% CI)** | **b (95% CI)** | **b (95% CI)** | **b (95% CI)** | **b (95% CI)** | **with age** |
| **Internalizing symptoms ^a^** |  |  |  |  |  |  |  |  |
| STAI-Trait anxiety | 197 |  | **0.71 (0.39, 1.03)** | **0.74 (0.32, 1.17)** | **0.79 (0.35, 1.22)** | **0.78 (0.33, 1.23)** | **0.49 (0.02, 0.96)** | 0.535 |
| EPQ-Psychoticism | 186 |  | 0.87 (-0.36, 2.10) | 1.38 (-0.27, 3.03) | 0.65 (-1.12, 2.42) | 0.12 (-1.76, 2.01) | 0.91 (-0.77, 2.59) | 0.686 |
| EPQ-Extraversion | 186 |  | -0.51 (-1.20, 0.17) | -0.68 (-1.71, 0.34) | -0.31 (-1.38, 0.75) | -0.72 (-1.79, 0.35) | -0.34 (-1.34, 0.66) | 0.889 |
| EPQ-Neuroticism | 185 |  | **1.62 (1.09, 2.15)** | **1.65 (0.89, 2.41)** | **1.58 (0.81, 2.34)** | **2.17 (1.35, 2.99)** | **1.24 (0.45, 2.03)** | 0.345 |
| EPDS Antenatal | 188 |  | **1.19 (0.64, 1.75)** | **1.26 (0.51, 2.00)** | **1.08 (0.28, 1.88)** | **1.69 (0.91, 2.47)** | **0.88 (0.09, 1.66)** | 0.328 |
| EPDS Antenatal (≥13) | 188 |  | **15.88 (8.66, 23.10)** | **18.40 (7.77, 29.03)** | **13.96 (3.41, 24.52)** | **24.61 (14.47, 34.76)** | 7.49 (-2.87, 17.84) | **0.008** |
| EPDS Postnatal | 349 |  | **0.83 (0.35, 1.31)** | **0.99 (0.41, 1.58)** | **1.03 (0.34, 1.71)** | **1.15 (0.44, 1.86)** | 0.05 (-0.69, 0.80) | **0.034** |
| EPDS Postnatal (≥13) | 349 |  | 6.01 (-1.63, 13.65) | 12.06 (2.91, 21.21) | 2.45 (-8.21, 13.10) | 6.71 (-4.76, 18.19) | -0.58 (-11.25, 10.10) | 0.161 |
| **Externalizing symptoms ^b^** |  |  |  |  |  |  |  |  |
| STAI-Trait anxiety | 196 |  | **0.69 (0.34, 1.04)** | **0.78 (0.35, 1.21)** | **0.80 (0.36, 1.23)** | **0.70 (0.21, 1.19)** | 0.29 (-0.21, 0.80) | 0.149 |
| EPQ-Psychoticism | 185 |  | -0.20 (-1.63, 1.22) | 1.05 (-0.65, 2.74) | -1.05 (-2.77, 0.67) | -1.54 (-3.58, 0.49) | -0.26 (-2.32, 1.80) | **0.044** |
| EPQ-Extraversion | 185 |  | -0.46 (-1.13, 0.22) | -0.35 (-1.23, 0.53) | **-1.01 (-1.93, -0.10)** | -0.54 (-1.60, 0.52) | 0.32 (-0.67, 1.32) | 0.135 |
| EPQ-Neuroticism | 184 |  | **1.72 (1.20, 2.25)** | **1.92 (1.25, 2.59)** | **1.58 (0.78, 2.39)** | **2.03 (1.20, 2.87)** | **1.28 (0.45, 2.11)** | 0.316 |
| EPDS Antenatal | 187 |  | **0.99 (0.42, 1.57)** | **1.01 (0.30, 1.72)** | **1.24 (0.51, 1.97)** | **1.25 (0.44, 2.06)** | 0.43 (-0.44, 1.31) | 0.222 |
| EPDS Antenatal (≥13) | 187 |  | 6.95 (-0.78, 14.68) | 8.82 (-0.65, 18.29) | 8.41 (-1.77, 18.58) | **14.35 (2.97, 25.72)** | -3.76 (-14.91, 7.39) | **0.014** |
| EPDS Postnatal | 347 |  | **0.98 (0.51, 1.45)** | **0.99 (0.43, 1.56)** | **1.46 (0.82, 2.10)** | **0.82 (0.06, 1.57)** | 0.20 (-0.52, 0.91) | **0.007** |
| EPDS Postnatal (≥13) | 347 |  | 6.80 (-0.30, 13.90) | **10.88 (2.29, 19.48)** | 8.59 (-0.98, 18.15) | 2.34 (-8.93, 13.61) | -3.05 (-13.69, 7.58) | 0.116 |
| **ADHD symptoms ^b^** |  |  |  |  |  |  |  |  |
| STAI-Trait anxiety | 196 |  | **0.45 (0.11, 0.79)** | **0.62 (0.17, 1.07)** | **0.56 (0.14, 0.99)** | 0.17 (-0.34, 0.68) | 0.04 (-0.43, 0.52) | 0.104 |
| EPQ-Psychoticism | 186 |  | 0.22 (-1.04, 1.48) | 0.60 (-0.99, 2.19) | 0.23 (-1.40, 1.86) | 0.07 (-1.93, 2.08) | -0.48 (-2.32, 1.37) | 0.824 |
| EPQ-Extraversion | 186 |  | -0.02 (-0.69, 0.65) | 0.51 (-0.42, 1.44) | **-0.96 (-1.81, -0.12)** | -0.01 (-1.07, 1.05) | 0.48 (-0.53, 1.48) | **0.004** |
| EPQ-Neuroticism | 185 |  | **1.14 (0.58, 1.70)** | **1.41 (0.67, 2.15)** | **1.17 (0.43, 1.91)** | 0.78 (-0.09, 1.64) | 0.81 (-0.04, 1.65) | 0.502 |
| EPDS Antenatal | 188 |  | 0.49 (-0.07, 1.05) | **0.85 (0.14, 1.55)** | 0.56 (-0.16, 1.28) | 0.06 (-0.77, 0.89) | -0.18 (-1.03, 0.68) | 0.120 |
| EPDS Antenatal (≥13) | 188 |  | 3.76 (-3.94, 11.46) | 6.54 (-3.36, 16.43) | 5.06 (-4.99, 15.10) | 0.25 (-10.61, 11.11) | -2.28 (-13.11, 8.54) | 0.317 |
| EPDS Postnatal | 349 |  | **0.63 (0.16, 1.10)** | **0.84 (0.24, 1.43)** | **0.76 (0.05, 1.46)** | 0.30 (-0.47, 1.06) | 0.31 (-0.39, 1.01) | 0.449 |
| EPDS Postnatal (≥13) | 349 |  | 6.10 (-0.83, 13.03) | **9.21 (0.57, 17.85)** | 8.94 (-1.29, 19.17) | 0.98 (-10.42, 12.38) | -0.13 (-10.43, 10.17) | 0.302 |

*Abbreviations*: ADHD: Attention Deficit Hyperactivity Disorder; EPDS: Edinburgh Postnatal Depression Scale; EPQ: Eysenck Personality Questionnaire; STAI: State-Trait Anxiety Inventory.

**^a^** Adjusted for child sex and exact age at assessment, maternal age, maternal smoking during pregnancy, preterm birth, breastfeeding duration, maternal education, paternal education, birth order and urban area of living.

**^b^** Adjusted for child sex and exact age at assessment, maternal age, maternal smoking during pregnancy, gestational age, breastfeeding duration, maternal education, paternal education, birth order and maternal working status.

Bold font indicates p < 0.05.

**Supplementary Table 4.** Age interaction and adjusted associations of perinatal maternal mental health and trajectories of internalizing, externalizing and ADHD symptoms from ages 4 to 15 years, mixed model analyses **after excluding children with learning disabilities or ADHD diagnosis**.

|  |  |  | **Across ages** | **4 years** | **6 years** | **11 years** | **15 years** | **p interaction** |
| --- | --- | --- | --- | --- | --- | --- | --- | --- |
|  | **N** |  | **b (95% CI)** | **b (95% CI)** | **b (95% CI)** | **b (95% CI)** | **b (95% CI)** | **with age** |
| **Internalizing symptoms ^a^** |  |  |  |  |  |  |  |  |
| STAI-Trait anxiety | 203 |  | **0.70 (0.37, 1.03)** | **0.70 (0.25, 1.15)** | **0.79 (0.34, 1.24)** | **0.75 (0.30, 1.20)** | **0.53 (0.04, 1.02)** | 0.685 |
| EPQ-Psychoticism | 194 |  | 1.04 (-0.24, 2.33) | 1.60 (-0.08, 3.28) | 0.71 (-1.11, 2.54) | 0.15 (-1.76, 2.05) | 1.26 (-0.50, 3.01) | 0.533 |
| EPQ-Extraversion | 194 |  | -0.52 (-1.21, 0.16) | -0.80 (-1.82, 0.23) | -0.01 (-1.06, 1.05) | -0.57 (-1.63, 0.50) | -0.71 (-1.69, 0.27) | 0.557 |
| EPQ-Neuroticism | 193 |  | **1.67 (1.10, 2.25)** | **1.44 (0.64, 2.24)** | **1.76 (0.96, 2.56)** | **2.25 (1.43, 3.07)** | **1.45 (0.62, 2.28)** | 0.320 |
| EPDS Antenatal | 194 |  | **1.33 (0.74, 1.93)** | **1.24 (0.45, 2.02)** | **1.40 (0.55, 2.25)** | **1.84 (1.04, 2.65)** | **1.00 (0.15, 1.85)** | 0.322 |
| EPDS Antenatal (≥13) | 194 |  | **16.57 (8.92, 24.22)** | **17.88 (6.41, 29.35)** | **15.74 (4.73, 26.75)** | **24.24 (13.98, 34.49)** | 9.19 (-1.94, 20.32) | **0.028** |
| EPDS Postnatal | 373 |  | **1.01 (0.55, 1.47)** | **1.10 (0.52, 1.68)** | **1.32 (0.64, 2.00)** | **1.37 (0.67, 2.07)** | 0.24 (-0.51, 0.99) | **0.030** |
| EPDS Postnatal (≥13) | 373 |  | **7.93 (0.51, 15.35)** | **12.44 (3.21, 21.67)** | 5.65 (-4.67, 15.97) | 9.07 (-2.46, 20.60) | 2.67 (-7.99, 13.32) | 0.439 |
| **Externalizing symptoms ^b^** |  |  |  |  |  |  |  |  |
| STAI-Trait anxiety | 202 |  | **0.68 (0.30, 1.06)** | **0.72 (0.27, 1.17)** | **0.75 (0.29, 1.21)** | **0.78 (0.27, 1.29)** | 0.34 (-0.19, 0.87) | 0.297 |
| EPQ-Psychoticism | 193 |  | 0.15 (-1.32, 1.61) | 1.46 (-0.25, 3.18) | -0.53 (-2.27, 1.21) | -1.59 (-3.69, 0.50) | -0.09 (-2.21, 2.02) | **0.027** |
| EPQ-Extraversion | 193 |  | -0.46 (-1.13, 0.21) | -0.33 (-1.19, 0.52) | -0.85 (-1.78, 0.09) | -0.46 (-1.53, 0.61) | -0.08 (-1.06, 0.90) | 0.562 |
| EPQ-Neuroticism | 192 |  | **1.74 (1.18, 2.30)** | **1.68 (0.96, 2.39)** | **1.72 (0.91, 2.53)** | **2.25 (1.38, 3.12)** | **1.47 (0.62, 2.32)** | 0.442 |
| EPDS Antenatal | 193 |  | **1.11 (0.49, 1.73)** | **0.99 (0.23, 1.75)** | **1.42 (0.64, 2.21)** | **1.52 (0.69, 2.35)** | 0.54 (-0.42, 1.50) | 0.106 |
| EPDS Antenatal (≥13) | 193 |  | 7.40 (-0.96, 15.76) | 7.47 (-2.62, 17.56) | 9.47 (-1.52, 20.46) | **15.71 (3.91, 27.50)** | -3.22 (-15.33, 8.90) | **0.013** |
| EPDS Postnatal | 371 |  | **1.17 (0.70, 1.64)** | **1.22 (0.66, 1.77)** | **1.56 (0.91, 2.22)** | **1.07 (0.29, 1.85)** | 0.46 (-0.28, 1.20) | **0.028** |
| EPDS Postnatal (≥13) | 371 |  | **10.70 (3.80, 17.60)** | **13.08 (4.38, 21.77)** | **14.01 (4.32, 23.70)** | 6.91 (-4.86, 18.68) | 2.43 (-8.35, 13.21) | 0.198 |
| **ADHD symptoms ^b^** |  |  |  |  |  |  |  |  |
| STAI-Trait anxiety | 202 |  | **0.43 (0.09, 0.77)** | **0.51 (0.07, 0.96)** | **0.57 (0.17, 0.98)** | 0.31 (-0.20, 0.82) | 0.13 (-0.35, 0.60) | 0.251 |
| EPQ-Psychoticism | 194 |  | 0.39 (-0.91, 1.68) | 1.08 (-0.51, 2.68) | 0.30 (-1.34, 1.95) | -0.33 (-2.32, 1.66) | -0.23 (-2.07, 1.62) | 0.599 |
| EPQ-Extraversion | 194 |  | 0.01 (-0.66, 0.68) | 0.57 (-0.35, 1.50) | **-0.83 (-1.66, -0.00)** | 0.05 (-1.00, 1.10) | 0.16 (-0.81, 1.12) | **0.014** |
| EPQ-Neuroticism | 193 |  | **0.97 (0.39, 1.54)** | **1.11 (0.34, 1.89)** | **1.04 (0.33, 1.75)** | 0.82 (-0.05, 1.68) | 0.71 (-0.18, 1.61) | 0.851 |
| EPDS Antenatal | 194 |  | 0.50 (-0.13, 1.13) | **0.84 (0.05, 1.62)** | 0.70 (-0.06, 1.46) | 0.26 (-0.60, 1.12) | -0.23 (-1.15, 0.68) | 0.067 |
| EPDS Antenatal (≥13) | 194 |  | 2.89 (-5.22, 10.99) | 6.72 (-3.68, 17.12) | 5.12 (-5.04, 15.27) | -0.25 (-10.90, 10.41) | -5.17 (-16.03, 5.69) | 0.093 |
| EPDS Postnatal | 373 |  | **0.75 (0.27, 1.23)** | **1.08 (0.48, 1.68)** | **0.88 (0.17, 1.58)** | 0.49 (-0.27, 1.26) | 0.22 (-0.48, 0.92) | 0.189 |
| EPDS Postnatal (≥13) | 373 |  | **7.55 (0.63, 14.46)** | **13.37 (4.39, 22.35)** | 10.18 (-0.09, 20.44) | 2.39 (-8.88, 13.66) | -1.91 (-11.84, 8.02) | 0.080 |

*Abbreviations*: ADHD: Attention Deficit Hyperactivity Disorder; EPDS: Edinburgh Postnatal Depression Scale; EPQ: Eysenck Personality Questionnaire; STAI: State-Trait Anxiety Inventory.

**^a^** Adjusted for child sex and exact age at assessment, maternal age, maternal smoking during pregnancy, preterm birth, breastfeeding duration, maternal education, paternal education, birth order and urban area of living.

**^b^** Adjusted for child sex and exact age at assessment, maternal age, maternal smoking during pregnancy, gestational age, breastfeeding duration, maternal education, paternal education, birth order and maternal working status.

Bold font indicates p < 0.05
